# Microsatellites in the Estrogen Receptor (*ESR1*, *ESR2*) and Androgen Receptor (*AR*) Genes and Breast Cancer Risk in African American and Nigerian Women

**DOI:** 10.1371/journal.pone.0040494

**Published:** 2012-07-11

**Authors:** Yonglan Zheng, Dezheng Huo, Jing Zhang, Toshio F. Yoshimatsu, Qun Niu, Olufunmilayo I. Olopade

**Affiliations:** 1 Center for Clinical Cancer Genetics and Global Health, Department of Medicine, The University of Chicago, Chicago, Illinois, United States of America; 2 Department of Health Studies, The University of Chicago, Chicago, Illinois, United States of America; IFOM, Fondazione Istituto FIRC di Oncologia Molecolare, Italy

## Abstract

Genetic variants in hormone receptor genes may be crucial predisposing factors for breast cancer, and microsatellites in the estrogen receptor (*ESR1*, *ESR2)* and androgen receptor (*AR*) genes have been suggested to play a role. We studied 258 African-American (AA) women with breast cancer and 259 hospital-based controls, as well as 349 Nigerian (NG) female breast cancer patients and 296 community controls. Three microsatellites, ESR1_TA, ESR2_CA and AR_CAG, in the *ESR1*, *ESR2* and *AR* genes, respectively, were genotyped. Their repeat lengths were then analyzed as continuous and dichotomous variables. Analyses of continuous variables showed no association with breast cancer risk in either AA or NG at ESR1_TA; AA cases had shorter repeats in the long allele of ESR2_CA than AA controls (Mann-Whitney *P*  = 0.036; logistic regression *P*  = 0.04, OR  = 0.91, 95% CI 0.83–1.00), whereas NG patients had longer repeats in the short allele than NG controls (Mann-Whitney *P*  = 0.0018; logistic regression *P*  = 0.04, OR  = 1.06, 95% CI 1.00–1.11); and AA cases carried longer repeats in the short allele of AR_CAG than AA controls (Mann-Whitney *P*  = 0.038; logistic regression *P*  = 0.03, OR  = 1.08, 95% CI 1.01–1.15). When allele sizes were categorized as dichotomous variables, we discovered that women with two long alleles of ESR2_CA had increased risk of breast cancer (OR  = 1.38, 95% CI 1.10–1.74; *P*  = 0.006). This is the first study to investigate these three microsatellites in hormonal receptor genes in relation to breast cancer risk in an indigenous African population. After adjusting for multiple-testing, our findings suggest that ESR2_CA is associated with breast cancer risk in Nigerian women, whereas ESR1_TA and AR_CAG seem to have no association with the disease among African American or Nigerian women.

## Introduction

Breast cancer is a major global problem, as it is one of the most commonly diagnosed cancers among women. In the United States, breast cancer is the first leading and the second fatal cancer among female cancer patients [1], while in western Africa, breast cancer has the second highest incidence and death rates [2]. Given the complex and comprehensive nature of breast cancer, it is reasonable to hypothesize that a joint effect of genetic factors as well as endogenous and exogenous hormonal and other environmental factors could contribute to an increased risk for breast cancer development.

Attention has long been paid to hormonal influences in breast cancer pathogenesis. Substantial evidence from both epidemiologic and experimental studies demonstrates a crucial role of hormones in the etiology of breast cancer. The indirect epidemiological support has come from the link between established reproductive breast cancer risk factors, such as early age at menarche and late age at menopause, and the exposures to estrogens and progesterone [3]. Consistent observations of higher levels of circulating estrogens and androgens in postmenopausal breast cancer women compared with controls provide the direct epidemiological evidence of hormones and breast cancer risk, whereas the link between sex steroids and premenopausal breast cancer risk still remains unclear [4]. Laboratory studies have showed that estrogens contribute to breast carcinogenesis via estrogen receptor (ER)-mediated cell proliferation, genotoxic effects of the metabolites, or the induction of aneuploidy [5]. Androgens have been suggested to influence breast cancer risk, either by directly binding to androgen receptors and either increasing or decreasing breast cell growth and proliferation, or by binding indirectly, through their conversion to estradiol or competitive binding to ER-α [6].

Microsatellites/short tandem repeats (STRs) are informative genetic markers in the human genome and they could have biological functions according to their locations, such as affecting protein coding (in coding regions), or regulating gene expression (in regulatory regions) [7]. Microsatellites in the estrogen receptor (*ESR1*, *ESR2)* and androgen receptor (*AR*) genes have been hypothesized to be predisposing factors for breast cancer but the mechanisms are unknown. A dinucleotide TA repeat polymorphism (ESR1_TA) is located in the promoter region of *ESR1* isoforms 1 and 2, as well as intron 1 and intron 2 of *ESR1* isoforms 3 and 4, respectively (6q25.1), while a polymorphic dinucleotide CA tandem repeat (ESR2_CA) is located in intron 5 of *ESR2* (14q23.2). To date, the functional property of these two microsatellites remains unknown, although gene expression could be modulated by such repeat nucleotide sequences [7]. AR (Xq12) is a ligand-dependent transcription factor, and its first exon contains a trinucleotide CAG repeat (AR_CAG) which has been of active interest, compared to ESR1_TA and ESR2_CA. AR_CAG encodes a polyglutamine (PolyGln) tract in the N-terminal transactivation domain of the AR protein, and it has been elucidated that PolyGln length inversely correlates with AR transcriptional competence [8]. Over the last decade, intensive association studies have been conducted to test the relationship between these three microsatellites and breast cancer risk in men and women, predominantly focusing on AR_CAG (Table 1). However, the results are controversial due to different study designs and different races/ethnicities of the study subjects, as a population-specific manner could exist. In addition, most of the previous study populations were Caucasians, some were Asians, and only one study population was African American (Table 1).

**Table 1:** Summary of the literature about ESR1_TA, ESR2_CA, and AR_CAG microsatellites and breast cancer risk.

Author, year	Study type	Major population	Affected/Unaffected	Sample notes	DNA	Major dichotomous repeat cutoff	Main findings
**ESR1_TA**							
Wedrén, 2004 [14]	Case-control	Swedish	1514/1514	Female, postmenopausal	Somatic, germline	14	Deviance from HWE
Iobagiu, 2006 [15]	Case-control	French	139/145	Female	Germline	19	No association
Anghel, 2006 [24]	Case-control	Romanian	153/318	Female	Somatic, germline	16, 23, 38 (sum)	Shorter repeats higher risk
Tsezou, 2008 [16]	Case-control	Greek	79/155	Female	Germline	15	No association
**ESR2_CA**							
Försti, 2003 [23]	Case-control	Finnish	219/248	Female cases and male controls	Germline	NA	No association
Iobagiu, 2006 [15]	Case-control	French	139/145	Female	Germline	22	No association
Anghel, 2006 [24]	Case-control	Romanian	153/318	Female	Somatic, germline	22, 23, 45 (sum)	Shorter repeats higher risk
Tsezou, 2008 [16]	Case-control	Greek	79/155	Female	Germline	20	Longer repeats less risk
**AR_CAG**							
Rebbec, 1999 [28]	Case-control	American	165/139	Female, *BRCA1* mutation carriers	Germline	28	Longer repeats higher risk and earlier diagnosis
Spurdle, 1999 [29]	Case-control	Australian	368/284	Female, <40 years	Germline	22	No association overall, or stratified by family history, ER and PR status
Dunning, 1999 [30]	Case-control	British	508/426	Female	Germline	21, 28	No association
Young, 2000 [31]	Case-control	Scotch	59/79	Male	Somatic, germline	NA	No association
Given, 2000 [32]	Case only	Irish	178/−	Female, ≤65 years	Germline	28	No association
Yu, 2000 [33]	Case only	Italian	133/−	Female	Somatic	21, 44 (sum)	Longer repeats associated with lower grade of tumors, fewer lymph node metastases and longer survival
Menin, 2001 [34]	Case only	Italian	101+50/−	Some *BRCA1/2* mutation carriers, 50 ovarian cancers	Germline	19, 23	No association
Kadouri, 2001 [35]	Case-control	Ashkenazi Jewish	152+166/36+152	Female, 188 *BRCA1/2* mutation carriers including ovarian cancers	Germline	28	No association
Giguèr, 2001 [36]	Case-control	French-Canadian	255/461	Female	Germline	20, 39 (sum)	Shoter repeats less risk, association stronger in postmenopausal women
Elhaji, 2001 [37]	Case-control	Canadian	114/248	Female, >40 years	Somatic, germline	26	Longer repeats higher risk in well and moderately differential tumors
Kristiansen, 2002 [38]	Case-control	Norwegian	216/575	Female, heterozygous cases for the CAG repeat	Germline	30	No association
Haiman, 2002 [39]	Case-control	European-American	727/969	Female, predominantly postmenopausal	Germline	22	No association overall, longer repeats associated with family history
Dagan, 2002 [40]	Case-control	Israeli Jewish	108+41/78	Female, *BRCA1/2* mutation carriers, 41 ovarian cancers	Germline	18, 19	Shorter repeats earlier onset
Suter, 2003 [41]	Case-control	European-American	524/461	Female, <45 years	Germline	22, 43 (sum)	Longer repeats higher risk, homozygous of CAG short allele associated with ever use of oral contraceptives
Syrjäkoski, 2003 [42]	Case only	Finnish	32/−	Male	Germline	NA	No association
Liede, 2003 [43]	Case-control	Filipino	299/229	Female	Germline	25	Longer repeats higher risk
MacLean, 2004 [44]	Case only	Australian	41/−	Male	Somatic	24	Longer repeats are more frequent in patients with grade I and II tumors, but not associated with age at diagnosis
Spurdle, 2004 [45]	Case-control	Australian & British	331+32/241	Female, *BRCA1/2* mutation carriers, 32 ovarian caners	Germline	28, 29	No association
Wang, 2005 [9]	Case-control	African-American	239/	Female	Germline	22	No association overall, longer repeats associated with family history
Iobagiu, 2006 [15]	Case-control	French	249139/145	Female	Germline	15	Shorter repeats higher risk
Anghel, 2006 [24]	Case-control	Romanian	153/318	Female	Somatic, germline	13, 16, 28 (sum)	Longer repeats higher risk
Cox, 2006 [27]	Case-control	European-American & European	95+376+669/540+674	Female	Germline	22, 23, 25, 27	No association
Slattery, 2007 [46]	Case-control	European-American & Hispanic-American	1,169+576/1,330+725	Female	Germline	22	No association
González, 2007 [47]	Case-control	Spanish	257/393	Female	Germline	22	Longer repeats higher risk
Wedrén, 2007 [48]	Case-control	Swedish	1,542/1,511	Female, postmenopausal	Somatic, germline	22, 20	No association overall
Tsezou, 2008 [16]	Case-control	Greek	79/155	Female	Germline	22	Longer repeats less risk
Wu, 2008 [49]	Case-control	Taiwanese	88/334	Female	Germline	22	Longer repeats associated with short duration of early estrogen exposure
Abbas, 2010 [50]	Case-control	Germany	3,149/5,489	Female	Germline	22	Shorter repeats associated with combined estrogen-progestagen therapy
Chintamani, 2010 [51]	Case-control	Indian	70/80	Female	Germline	NA	No association
Sakoda, 2010 [52]	Case-control	Chinese	614+467/879	Female, 467 subjects with fibrocystic conditions	Germline	22–24	Longer repeats associated with an increased risk of fibrocystic breast conditions
Hietala, 2010 [53]	htSNP/diplotype	Swedish	56/269	Female, 56 *BRCA1/2* mutation carriers and 269 young healthy subjects from breast cancer high-risk families	Germline	22	Neither individual htSNPs nor other diplotypes were significantly associated with androgen levels and did not tag for AR microsatellites
Rajender, 2010 [54]	Case-control	Indian	747/661	Female	Germline	NA	No association overall, no significant difference between pre- and post-menopausal cases
Mehdipour, 2010 [55]	Case-control	Iranian	500/432	Female	Germline	22	Longer repeats higher risk, but reduce the risk in subjects with positive family history
Gilbert, 2011 [56], [57]	Case-control	Egyptian	44/43	Male	Somatic, germline	14, 22	No association

In the present study, we aimed to investigate whether ESR1_TA, ESR2_CA, and AR_CAG could be breast cancer susceptibility markers in African American (AA) and Nigerian (NG) women. We used a case-control study design with 258 AA cases and 259 AA controls, together with 349 NG cases and 296 NG controls.

## Materials and Methods

### Ethics Statement

Informed consent was written and obtained from all participants. This study was approved by the Institutional Review Boards of the University of Chicago and the University of Ibadan.

### Study Populations

The Chicago Cancer Prone Study (CCPS): CCPS is an ongoing hospital-based case-control study aimed at understanding the genetic basis of young-onset breast cancer. Histologically confirmed breast cancer patients were recruited through the Cancer Risk Clinic at the University of Chicago. Cancer-free healthy controls were gender- and age-matched with cases and enrolled from individuals who visited the same hospital (Translational Research Initiative in the Department of Medicine, TRIDOM). Included in this study were 258 AA cases (age of onset  = 44.1±10.0 years [mean ± SD]) and 259 AA controls (age  = 46.8±10.9 years).

The Nigerian Breast Cancer Study (NBCS): NBCS is an ongoing case-control study of breast cancer in Ibadan, Nigeria initiated in 1998. Breast cancer patients were recruited at the University College Hospital, Ibadan. Cancer-free healthy controls were randomly collected from a community adjoining the hospital, gender- and age-matched to the cases in the present study. The majority of the study subjects are Yoruban from Ibadan. NBCS contributed 349 NG cases (age of onset  = 47.5±11.7 years) and 296 NG controls (age  = 41.5±14.1 years) to this study.

Unfortunately, estrogen receptor (ER) status was available for only a small subset of the breast cancer cases: 88 AA ER+ cases, 86 AA ER- cases, 23 NG ER+ cases, and 48 NG ER- cases (Table S1 and Table S2).

### Sample Quality Control

Genomic DNA was extracted from whole blood and evaluated for integrity by electrophoresis on agarose gel. Double-strand DNA was quantitated using Quant-iT™ PicoGreen dsDNA kit (Invitrogen, CA, USA) and then quantified on Infinite® 200 PRO NanoQuant (Tecan, Männedorf, Switzerland), according to the manufacturer’s instructions. To examine the potential sample contamination and gender discrepancy, samples were amplified by polymerase chain reaction (PCR) using AmpFℓSTR® Identifiler® PCR Amplification kit (Applied Biosystems, CA, USA) and the PCR products were analyzed on Applied Biosystems 3130 DNA Analyzer (Applied Biosystems, CA, USA). DNA fragment data were collected and then individually checked using GeneMapper® software (Applied Biosystems, CA, USA), according to the manufacturer’s protocol.

### Determination of Microsatellite Allele Sizes

Primers were designed to amplify the fragments encompassing microsatellites ESR1_TA, ESR2_CA and AR_CAG present in *ESR1*, *ESR2* and *AR*, respectively: ESR1_TA_F: 5′-6FAM-AGACGCATGATATACTTCACC-3′, ESR1_TA_R: 5′-GTTCACTTGGGCTAGGATAT-3′ (amplicon: chr6:152127623–152127811, GRCh37/hg19); ESR2_CA_F: 5′-HEX-AACAAAATGTTGAATGAGTGGG-3′, ESR2_CA_R: 5′-GGTAAACCATGGTCTGTACC-3′ (amplicon: chr14:64720235–64720395, GRCh37/hg19); AR_CAG_F: 5′-HEX-TCCAGAATCTGTTCCAGAGCGTGC-3′, AR_CAG_R: 5′-GCTGTGAAGGTTGCTGTTCCTCAT-3′ (amplicon: chrX:66765056–66765343, GRCh37/hg19). PCR was performed using PCRx Enhancer System (Invitrogen, CA, USA) with conditions as follows: initial denaturation at 95°C for 5 min, followed by 35 amplification cycles at 95°C for 30 sec, annealing at 55°C for 50 sec and 72°C for 50 sec, followed by a final extension step at 72°C for 10 min. Fluorescently labeled fragments generated by PCR were run on Applied Biosystems 3130 DNA Analyzer and the repeat lengths were subsequently checked and assigned in GeneMapper® software. Genotypes were determined by two independent investigators who were blinded to subject disease status and all clinical information. Multiple homozygous subjects for each individual microsatellite were randomly chosen from the same studied populations and sequenced. Repeat lengths read by sequencing were successfully assigned to corresponding peak positions determined by fluorescence-based genotyping. Ninety-six samples were randomly selected and repeated to test the assay reproducibility.

### Post-genotyping Quality Measurements

Tests of conformance to Hardy-Weinberg genotypic expectations were carried out with Genepop v4.1 (available at http://genepop.curtin.edu.au/). In addition, we checked the microsatellite data by Micro-Checker v2.2.3, which was designed to identify genotyping errors due to an excess of homozygotes caused by non-amplified alleles (null alleles), stutter peaks, or short allele dominance (large allele dropout) (available at http://www.microchecker.hull.ac.uk/). Genotypes that did not deviate from Hardy-Weinberg Equilibrium (HWE) were eligible for statistical association analyses.

### Statistical Analysis

The repeat lengths for ESR1_TA, ESR2_CA and AR_CAG were classified as continuous and categorical variables, separately. For the continuous variable analysis, two alleles of a single microsatellite carried by each woman were assigned as the short allele (S) and the long allele (L), according to the smaller and larger allele sizes determined, respectively. For homozygotes, two alleles are identical in peak positions; they were assigned as one S and one L, and later included in both the short and long allele analyses. For each microsatellite, the repeat lengths (mean ± standard deviation [SD]) were calculated under three categories: S, L, and the average of them. Wilcoxon rank-sum (Mann-Whitney) test was applied to compare the distributions of the repeat lengths, between case and control groups of AA and NG. Van Elteren’s test which is a stratified version of Wilcoxon rank-sum test was used to compare the repeat length in the pooled AA and NG sample set. Odds ratios (ORs) were also calculated by logistic regression analysis controlling for ascertainment, and with 95% confidence interval (CI) for three microsatellites, in AA, NG, and AA + NG. In addition, in order to avoid too-strong assumptions allele sizes were classified into dichotomous groups. Given that there is no *a priori* cut-off point applicable to distinguish satisfactorily the short and long alleles for ESR1_TA, ESR2_CA or AR_CAG, the mean repeat lengths of these three microsatellites in control groups were chosen as cut-off points, respectively. The cut-off limits were 18 (S: <18, L: ≥18) for ESR1_TA, 23 (S: <23, L: ≥23) for ESR2_CA, and 20 (S: <20, L: ≥20) for AR_CAG (see Results and Table 2). The comparisons of genotype distributions between case and control groups were then performed in unconditional logistic regression models for AA and NG separately and as pooled samples, based on the categorical variables corresponding to the three microsatellites. Further, a cut-off point of 22 for AR_CAG was also chosen in an attempt to allow direct comparison of our results to the data previously reported in the literature. Moreover, since Wang and colleagues have conducted the association test between AR_CAG and breast cancer risk in AA using a dichotomized cut-off point of 22 [9], we also compared our data to theirs to see whether the findings were consistent in the same ethnic population. Moreover, we conducted a similar analysis for the ER status of breast cancer cases. Given the limited sample size, we combined AA and NG for both continuous and categorical variable analyses stratified by ER status (Table S1 and Table S2). The statistical analysis was conducted using Stata 11.1 software (StataCorp, TX, USA) and SAS 9.2 package (SAS Institute, NC, USA). All statistical tests were two-sided. Given three tested loci and two populations, the number of multiple-testing was 6. Thus, the significant threshold was set as 0.05/6 = 0.0083.

**Table 2:** Continuous variable analysis of ESR1_TA, ESR2_CA, and AR_CAG alleles and breast cancer risk in African American and Nigerian women.

		Repeat length, mean ± SD				Mann-Whitney *P* 2			Logistic regression *P* 2, OR (95% CI)		
Microsatellite	Allele^1^	AA Case	AA Control	NG Case	NG Control	AA	NG	AA + NG^3^	AA	NG	AA + NG
ESR1_TA	S	15.32±2.93	15.06±2.78	15.01±2.77	14.78±2.66	0.51	0.23	0.18	0.31, 1.03 (0.97–1.10)	0.31, 1.03 (0.97–1.09)	0.15, 1.03 (0.99–1.08)
	L	19.31±3.58	19.66±3.63	19.36±3.69	19.47±3.30	0.21	0.97	0.42	0.26, 0.97 (0.93–1.02)	0.69, 0.99 (0.95–1.04)	0.29, 0.98 (0.95–1.02)
	Ave.	17.31±2.86	17.36±2.78	17.18±2.76	17.13±2.53	0.85	0.74	0.91	0.84, 0.99 (0.94–1.06)	0.79, 1.01 (0.95–1.07)	0.96, 1.00 (0.96–1.04)
ESR2_CA	S	21.36±2.52	21.27±3.03	21.52±3.05	21.02±2.94	0.72	**0.0018**	0.037	0.71, 1.01 (0.95–1.08)	0.04, 1.06 (1.00–1.11)	0.07, 1.04 (1.00–1.08)
	L	23.85±1.76	24.20±2.05	24.17±2.07	23.95±2.02	0.036	0.11	0.84	0.04, 0.91 (0.83–1.00)	0.17, 1.06 (0.98–1.14)	0.78, 0.99 (0.94–1.05)
	Ave.	22.61±1.74	22.74±2.13	22.84±2.10	22.49±2.05	0.15	**0.0047**	0.26	0.45, 0.97 (0.88–1.06)	0.03, 1.09 (1.01–1.17)	0.24, 1.04 (0.98–1.10)
AR_CAG	S	18.35±2.60	17.83±2.77	17.78±2.35	17.73±2.30	0.038	0.82	0.12	0.03, 1.08 (1.01–1.15)	0.81, 1.01 (0.94–1.08)	0.08, 1.04 (1.00–1.09)
	L	21.73±2.88	21.78±2.99	21.26±2.70	21.38±2.91	0.83	0.62	0.61	0.84, 0.99 (0.94–1.05)	0.58, 0.99 (0.93–1.04)	0.59, 0.99 (0.95–1.03)
	Ave.	20.04±2.38	19.81±2.42	19.52±2.11	19.56±2.22	0.50	0.98	0.64	0.26, 1.04 (0.97–1.12)	0.82, 0.99 (0.92–1.07)	0.53, 1.02 (0.97–1.07)

1 S: Short allele; L: Long allele; Ave.: average repeat length of short and long alleles.

2 *P* values <0.0083 were bolded.

3 Van Elteren’s test.

## Results

### Genotyping Quality

No samples were detected to have contamination or gender issues, no discrepancy of allele calling was found between two investigators, and genotypes of 96 repeated samples were 100% consistent with previous determinations. Genotyping call rates were 99.8%, 100%, and 100% for ESR1_TA, ESR2_CA, and AR_CAG, respectively, in AA; genotyping call rates were 99.5%, 100%, and 99.8% for ESR1_TA, ESR2_CA, and AR_CAG, respectively, in NG. Calculations from Genepop and Micro-Checker showed that genotypes for all three microsatellites were in HWE and no potential genotyping errors were detected.

### Distribution of ESR1_TA Alleles in Cases and Controls

**Figure 1:**
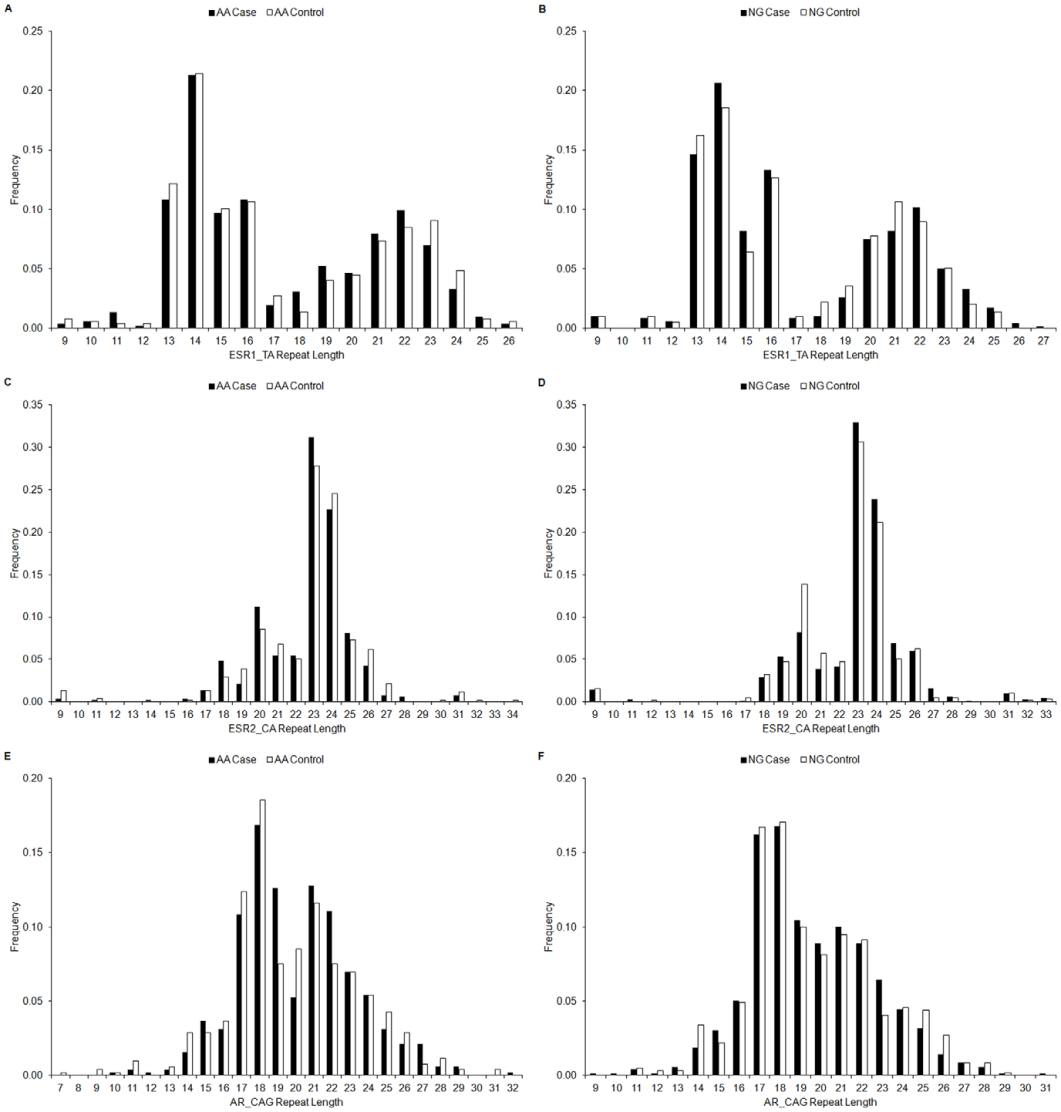
Allele distributions of ESR1_TA, ESR2_CA, and AR_CAG in AA and NG. (A) ESR1_TA allele distribution in AA. (B) ESR1_TA allele distribution in NG. (C) ESR2_CA allele distribution in AA. (D) ESR2_CA allele distribution in NG. (E) AR_CAG allele distribution in AA. (F) AR_CAG allele distribution in NG.

The repeat length of ESR1_TA ranged from 9 to 26 (S: 9–24; L: 9–26) in AA cases and 9 to 26 (S: 9–24; L: 13–26) in AA controls, whereas the range was 9 to 27 (S: 9–23; L: 13–27) in NG cases and 9 to 25 (S: 9–23; L: 13–25) in NG controls. The repeat lengths (mean ± SD) of ESR1_TA were 17.31±2.86 and 17.36±2.78 in AA cases and controls, respectively; repeat lengths were 17.18±2.76 and 17.13±2.53 in NG cases and controls, respectively (Table 2, Figure 1).

No significant difference in the distribution of ESR1_TA repeat length was observed between cases and controls in AA or NG, when the continuous variable analysis was performed classifying allele sizes to S, L, and Ave. (Table 2). We also dichotomized the repeat length based on the cut-off point of 22, which was the mean of repeat length in AA and NG controls. There was no significant association in the ESR1_TA genotype distribution in either AA or NG. Instead, we found a *P* value of 0.039 in logistic regression in the pooled sample set of AA + NG, but it did not retain significance after correction for multiple-testing (Table 3).

**Table 3:** Categorical variable analysis of ESR1_TA, ESR2_CA, and AR_CAG alleles and breast cancer risk in African American and Nigerian women.

			AA				NG				AA + NG	
Microsatellite	Dichotomous cut-off	Genotype^1^	Case, n (%)	Control, n (%)	OR (95% CI)	Logistic regression *P*	Case, n (%)	Control, n (%)	OR (95% CI)	Logistic regression *P* 2	OR (95% CI)	Logistic regression *P* 2
ESR1_TA	<18 vs. ≥18	SS	90 (35.0)	84 (32.4)	1.0 (ref.)		123 (35.2)	88 (30.0)	1.0 (ref.)		1.0 (ref.)	
		SL	115 (44.8)	138 (53.3)	0.78 (0.53–1.15)		173 (49.6)	164 (56.0)	0.75 (0.53–1.07)		0.77 (0.59–0.99)	
		LL	52 (20.2)	37 (14.3)	1.31 (0.78–2.20)		53 (15.2)	41 (14.0)	0.92 (0.57–1.51)		1.09 (0.77–1.56)	
		Total	257	259		0.089	349	293		0.26		0.039
		LL vs. SS + SL			1.52 (0.96–2.42)	0.075			1.10 (0.71–1.71)	0.67	1.29 (0.93–1.77)	0.12
		SL + LL vs. SS			0.89 (0.62–1.28)	0.53			0.79 (0.57–1.10)	0.16	0.83 (0.65–1.07)	0.15
ESR2_CA	<23 vs. ≥23	SS	26 (10.1)	22 (8.5)	1.0 (ref.)		29 (8.3)	28 (9.5)	1.0 (ref.)		1.0 (ref.)	
		SL	111 (43.0)	113 (43.6)	0.83 (0.44–1.55)		125 (358)	148 (50.0)	0.82 (0.46–1.44)		0.82 (0.54–1.25)	
		LL	121 (46.9)	124 (47.9)	0.83 (0.44–1.54)		195 (55.9)	120 (40.5)	1.57 (0.89–2.77)		1.17 (0.77–1.78)	
		Total	258	259		0.82	349	296		**0.0004**		0.016
		LL vs. SS + SL			0.96 (0.68–1.36)	0.82			1.86 (1.36–2.54)	**<0.001**	1.38 (1.10–1.74)	**0.006**
		SL + LL vs. SS			0.83 (0.46–1.50)	0.54			1.15 (0.67–1.99)	0.61	0.99 (0.66–1.48)	0.97
AR_CAG	<20 vs. ≥20	SS	66 (25.6)	61 (23.6)	1.0 (ref.)		101 (29.0)	87 (29.5)	1.0 (ref.)		1.0 (ref.)	
		SL	125 (48.4)	138 (53.3)	0.84 (0.55–1.28)		180 (51.7)	154 (52.2)	1.01 (0.70–1.44)		0.93 (0.71–1.22)	
		LL	67 (26.0)	60 (23.2)	1.03 (0.63–1.69)		67 (19.3)	54 (18.3)	1.07 (0.68–1.69)		1.06 (0.76–1.48)	
		Total	258	259		0.54	348	295		0.95		0.67
		LL vs. SS + SL			1.16 (0.78–1.74)	0.46			1.06 (0.72–1.58)	0.76	1.11 (0.84–1.48)	0.46
		SL + LL vs. SS			0.90 (0.60–1.34)	0.59			1.02 (0.73–1.44)	0.90	0.97 (0.75–1.25)	0.80
AR_CAG	<22 vs. ≥22	SS	124 (48.1)	127 (49.0)	1.0 (ref.)		192 (55.2)	155 (52.5)	1.0 (ref.)		1.0 (ref.)	
		SL	102 (39.5)	110 (42.5)	0.95 (0.66–1.37)		130 (37.4)	122 (41.4)	0.86 (0.62–1.19)		0.90 (0.70–1.15)	
		LL	32 (12.4)	22 (8.5)	1.49 (0.82–2.71)		26 (7.5)	18 (6.1)	1.17 (0.62–2.21)		1.33 (0.86–2.05)	
		Total	258	259		0.33	348	295		0.52		0.21
		LL vs. SS + SL			1.53 (0.86–2.70)	0.15			1.24 (0.67–2.31)	0.49	1.39 (0.91–2.12)	0.13
		SL + LL vs. SS			1.04 (0.74–1.47)	0.83			0.90 (0.66–1.23)	0.51	0.96 (0.76–1.21)	0.73

1 S: Short allele; L: Long allele.

2 *P* values <0.083 were bolded.

### Distribution of ESR2_CA Alleles in Cases and Controls

The repeat length of ESR2_CA ranged from 9 to 31 (S: 9–26; L: 18–31; average [Ave.] 22.61±1.74) in AA cases and 9 to 34 (S: 9–26; L: 18–34; Ave. 22.74±2.13) in AA controls, whereas it was 9 to 33 (S: 9–29; L: 19–33; Ave. 22.84±2.10) in NG cases and 9 to 33 (S: 9–26; L: 19–33; Ave. 22.49±2.05) in NG controls (Table 2, Figure 1).

When the repeat lengths of both ESR2_CA alleles were analyzed as continuous variables, AA cases appeared to have shorter L than AA controls (Mann-Whitney *P*  = 0.036; logistic regression *P*  = 0.04, OR  = 0.91, 95% CI 0.83–1.00), whereas NG patients had longer S than NG controls (Mann-Whitney *P*  = 0.0018; logistic regression *P*  = 0.04, OR  = 1.06, 95% CI 1.00–1.11) (Table 2). Thus, it turned out that L was the protective allele in AA and S was the risk allele in NG. In addition, it revealed that longer average repeat length of the ESR2_CA alleles was significantly more associated with breast cancer in NG (Mann-Whitney *P*  = 0.0047; logistic regression *P*  = 0.03, OR  = 1.09, 95% CI 1.01–1.17) (Table 2). According to the categorical repeat length cut-off of 23, comparisons of the ESR2_CA genotypes between case and control groups showed no significance in either AA or AA + NG, but there was significance in NG (*P*  = 0.0004) (Table 3). NG individuals with SS and SL genotypes (enrichment of the risk allele, S) had significantly increased risk of developing breast cancer compared to the ones with LL genotype (*P*<0.001, OR  = 1.86, 95% CI 1.36–2.54) (Table 3). Similarly, the trend was also observed in AA + NG (*P*  = 0.006, OR  = 1.38, 95% CI 1.10–1.74) (Table 3).

### Distribution of AR_CAG Alleles in Cases and Controls

The microsatellite AR_CAG located in exon 1 of the *AR* gene has repeat length ranging from 10 to 32 (S: 10–26; L: 15–32; Ave. 20.04±2.38) in AA cases and 7 to 31 (S: 7–25; L: 14–31; Ave. 19.81±2.42) in AA controls, whereas it is 9 to 31 (S: 9–25; L: 15–31; Ave. 19.52±2.11) in NG cases and 11 to 29 (S: 11–25; L: 14–29; Ave. 19.56±2.22) in NG controls (Table 2, Figure 1).

No statistical significance was found between AA patients and controls in the analysis of continuous variables for the distribution of AR_CAG repeat polymorphism. AA cases carried longer S of AR_CAG than AA controls (Mann-Whitney *P*  = 0.038; logistic regression *P*  = 0.03, OR  = 1.08, 95% CI 1.01–1.15). No statistically significant signal was obtained in either NG or AA + NG. In addition, we applied the dichotomous cut-offs of 20 and 22 for AR_CAG and then conducted genotypic logistic regression analysis. The results of this study provided no evidence that the AR_CAG genotypes can significantly influence the risk for breast cancer, in any populations (Table 3). Furthermore, the combined data set from us and Wang *et al.* [9] showed that there was no association of AR_CAG genotype and breast cancer risk in AA (SL + LL vs. SS, *P*  = 0.61, OR  = 1.07, 95% CI 0.82–1.38) (Table 4).

**Table 4:** Association between AR_CAG (categorical cut-off  = 22) and breast cancer in African American women.

	AA (the present study)			AA (Wang, 2005 [9])			AA (combined)			
Genotype^1^	Case, n (%)	Control, n (%)	OR (95% CI)	Case, n (%)	Control, n (%)	OR (95% CI)	Case, n (%)	Control, n (%)	OR (95% CI)	*P*
SS	124 (48.1)	127 (49.0)	1.0 (ref.)	145 (60.7)	156 (62.7)	1.0 (ref.)	269 (54.1)	283 (55.7)	1.0 (ref.)	0.61
SL + LL	134 (51.9)	132 (51.0)	1.04 (0.74–1.47)	94 (39.3)	93 (37.3)	1.09 (0.75–1.57)	228 (45.9)	225 (44.3)	1.07 (0.82–1.38)	

1 S: Short allele; L: Long allele.

## Discussion

Breast cancer is a hormone-dependent malignancy and cumulative exposure to sex hormones has been proposed to be linked to the development of breast cancer. Microsatellites ESR1_TA and ESR2_CA in the *ESR1* and *ESR2* genes, respectively, have also been reported to be associated with other diseases such as bone mineral density [10], osteoarthritis [11], and endometriosis [12]. Similarly, PolyGln tract of *AR* (encoded by AR_CAG) has been reported to be associated with susceptibility to a number of human diseases, such as prostate cancer, male infertility, cryptorchidism, hirsutism, Spinal and Bulbar Muscular Atrophy, and Kennedy’s disease, among others [8]. The ascertainments of ESR1_TA, ESR2_CA and AR_CAG in breast cancer have also been of scientific interest, especially on AR_CAG (Table 1). Nonetheless, the findings were not consistent with the AR_CAG genetic association studies that have been widely conducted in different racial/ethnic populations, except in those studying populations of African ancestry. In the present study, we targeted these three microsatellites and tested for germline susceptibility to female breast cancer, in two populations of African descent that were historically understudied for breast cancer genetics: African Americans and Nigerians. ESR1_TA, ESR2_CA and AR_CAG were genotyped in 1,162 female individuals consisting of 258 AA breast cancer cases, 259 AA controls, 349 NG breast cancer patients, and 296 NG controls. We then compared the repeat lengths of these three microsatellites between case and control groups using statistical methods considering continuous or categorical variables. With 607 case patients and 555 controls, the current study provides 80% power at an alpha level of 0.05 to detect an OR of 1.39 if we assume that the probability of AR_CAG long alleles greater than 22 is 0.5, under a dominant genetic model according to previous study. For ESR1_TA and ESR2_CA, the study provides 80% power at an alpha level of 0.05 to detect an OR of 1.44 under a recessive genetic model or 1.49 under a dominant genetic model if we categorize these two microsatellites biomarkers according to the median. The power for detecting case-control difference is higher if we analyze these three microsatellites biomarkers as continuous variables.

Analyzing each individual allele of a microsatellite is a way to examine its genetic/biological function. However, it is not easy to test whether an effect from a combination of alleles exists, and which alleles are actually involved. The combination could rely on repeat length (joint effect from particular alleles of repeat-length-dependent functional property), or allele frequency (quantitative-like effect from any functional alleles reaches a threshold). It therefore creates too many assumptions and comparisons, especially when the function (if any) of exact allele(s) of a microsatellite remains unclear. Previous studies applied either the mean repeat length as a cut-off, or a cut-off previously reported, sometimes even ignoring the allele distribution differences among different ethnic populations. We chose to set the mean repeat length as a cut-off, because it divides the distribution of microsatellite repeat lengths in approximately in half, maintains adequate numbers in each allele category, and therefore provides the greatest power to detect statistically significant differences between cases and controls.

Overall, the allele distributions of each individual microsatellite between AA and NG are similar (Figure 1). The allele distribution of AR_CAG in our AA is comparable to other AA cohorts [9]. Additionally, the spectrum of AR_CAG in the present study is consistent with previous observations that Africans have the shortest AR_CAG repeats, Asians bear the longest ones, while Caucasians and Mexican Americans are in the middle [13].

With the cut-off of ESR1_TA repeat length defined as 18, we found a *P* value of 0.039 in logistic regression in AA + NG, but not in AA or NG; however, this *P* value did not reach the significant threshold of 0.0083. Additionally, no statistical significance was observed in continuous variable analysis. It seems that the risk impact of ESR1_TA in breast cancer is weak or is very likely to be absent in AA and NG. A large case-control study of *ESR1* haplotype and postmenopausal breast cancer risk in Sweden has been carried out, but the ESR1_TA genotypes distribution deviated from HWE [14]. Studies in French and Greek populations suggested that ESR1_TA does not contribute to the development of breast cancer in women [15], [16].

In recent years, many genome-wide association studies (GWAS) have been conducted to search for breast cancer susceptibility loci. One of the GWAS hits, 6q25.1 (*CCDC170*-*ESR1* region), was identified in a Chinese population [17]. The most significant single nucleotide polymorphism (SNP), rs2046210, is located 3′ downstream (6kb away) of *CCDC170* and 5′ upstream (63 kb away) of the nearest *ESR1* isoform 4, a region covered by a single linkage disequilibrium (LD) block. This SNP has been replicated in Chinese and Japanese populations [18] but not in African Americans [18]–[21]. Furthermore, fine-mapping studies suggested that rs9397435 (2.9 kb away from rs2046210) could confer risk to all three populations in women of Asian, European, and African origin [22]. These two SNPs are 119 kb away from ESR1_TA which is located in another LD block. Thus, the role of germline genetic variants of *ESR1* in breast cancer etiology herein remains to be further clarified and explored. To date, neither *ESR2* nor *AR* genes has been identified by breast cancer GWAS.

For ESR2_CA, our analysis of continuous variables showed that AA cases had shorter L than AA controls (Mann-Whitney *P*  = 0.036; logistic regression *P*  = 0.04, OR  = 0.91, 95% CI 0.83–1.00). Thus, L turned out to be the protective allele in AA. However, the association results were negative when considering multiple-testing correction. So, the inherited predisposition of ESR2_CA to breast cancer in AA women was not fully supported. On the other hand, NG patients appeared to have longer S than NG controls (Mann-Whitney *P*  = 0.0047; logistic regression *P*  = 0.03, OR  = 1.09, 95% CI 1.01–1.17), namely, S was the risk allele in NG. In the logistic regression analysis using the categorical cut-off of 23, ESR2_CA genotype overall distribution in cases and controls showed no significance in either AA or AA + NG, but in NG (*P*  = 0.0004). When comparing LL vs. SS + SL, statistical significances were also obtained in NG (*P*<0.001, OR  = 1.86, 95% CI 1.36–2.54) and AA + NG (*P*  = 0.006, OR  = 1.38, 95% CI 1.10–1.74). Although the association of ESR2_CA and breast cancer risk has not been reported in Finnish [23] or French populations [15], shorter repeats of ESR2_CA has been found to be associated with higher breast cancer risk in Romanians [24], while longer repeats has been linked to less breast cancer risk in Greeks [16]. Our data were in line with the above observations in Romanians and Greeks.

The reversed association directions of ESR2_CA observed in our AA and NG cohorts, could be explained as a flip-flop phenomenon which has been frequently observed across different or even the same ethnic groups [25]. If a genetic variant is a functional causal variant (e.g. a confirmed missense mutation or splicing variant), its risk allele is supposed to be the same in different association tests, theoretically; if a flip-flop association occurs for a variant with unknown function (e.g. ESR2_CA or an intergenic SNP), it could indicate a false-positive or a true association – different risk alleles from different study populations tag/capture the same risk allele of another genuine causal variant in LD. In addition to different LD architectures, flip-flop associations could also be attributable to sample heterogeneity, sampling variations, multilocus interactions, and gene-environment interactions, across different racial/ethnic populations. Although African Americans and Nigerians share ancestral origin, they have differences in life style, environmental exposure, mutation selection balance, local recombination rate, LD pattern, among others. To the best of our knowledge, this is the first reported association of ESR2_CA and breast cancer risk in women of African ancestry; its flip-flop associations underscore the need for further and deeper validation in the same or similar populations.

Among the three microsatellites examined, AR_CAG is the most commonly tested one in the literature (Table 1). In the analysis of continuous variables, it showed that AA cases carried longer S of AR_CAG than AA controls (Mann-Whitney *P*  = 0.038; logistic regression *P*  = 0.03, OR  = 1.08, 95% CI 1.01–1.15), but no statistical significance was obtained after adjusting for multiple-testing. Furthermore, we did not gain any significant indications from logistic regression analysis when a dichotomized cut-off of 20 was applied. Wang et al. have genotyped AR_CAG in 239 AA breast cancer women and 249 controls and found no significant association between AR_CAG and overall breast cancer risk using a cut-off of 22 AR_CAG repeats [9]. Nonetheless, longer AR_CAG repeats were found to be associated with increased risk in AA women with a first-degree family history of breast cancer [9]. In order to compare our results to theirs, we also classified the AR_CAG repeat lengths using the same cut-off of 22. Our own data together with the results from the combined data set revealed no positive association between AR_CAG and breast cancer risk in AA women. We were unable to perform statistical comparisons in women with a first-degree family history of breast cancer, due to the very limited number of such individuals in the present study. Based on the results above, we can only cautiously draw a conservative conclusion that AR_CAG seems to influence the overall breast cancer risk weakly at most in AA, though it might affect the disease susceptibility to women having family history of breast cancer.

It is noteworthy that there have been divergent findings about the AR_CAG and breast cancer risk (Table 1). One possible explanation is that the previous studies applied different study designs (case-control study vs. case-only study), populations (Caucasians, Asians, etc.), subjects’ gender (female vs. male), sample size, DNA (germline vs. somatic), or dichotomous repeat length cut-offs. In addition, X-inactivation has been suspected to bias risk estimates, since *AR* is located on the X chromosome. However, it has been reported that the short and long AR_CAG alleles were subjected to skewed inactivation with similar frequency [26], and it has been suggested that X-inactivation might not be responsible to the estimation bias due to its early occurrence in the embryo and later in the different lobes within the same breast [27].

Since it has become clear in recent years that breast cancer genetic susceptibility is subtype specific, we conducted similar subset analysis stratified by ER status but found no significance (Table S1 and Table S2). It is worth noting that we were unable to obtain sufficient statistical power for the subtype analysis due to the limited sample size. Our work is a retrospective study primarily designed for overall breast cancer risk. In addition, doing immunohistochemistry work in Nigeria is a challenge because scientific and medical support is still an emergent need there. Future investigations are warranted to determine the risk conferred by these three microsatellites in breast cancer patients classified by subtypes.

To our knowledge, there have been only three previous reports investigating the association between all the three microsatellites (ESR1_TA, ESR2_CA, and AR_CAG) and risk of breast cancer in women [15], [16], [24], and the present study is the first to evaluate the risk impact of these microsatellites in hormonal receptor genes and the germline susceptibility to breast cancer in an indigenous African population. The capability of these three microsatellites to estimate breast cancer risk requires further replications in larger and more diverse populations.

## Supporting Information

Table S1 **Continuous variable analysis of ESR1_TA, ESR2_CA, and AR_CAG alleles and breast cancer risk stratified by ER status in African American and Nigerian women.**
(DOC)

Table S2 **Categorical variable analysis of ESR1_TA, ESR2_CA, and AR_CAG alleles and breast cancer risk stratified by ER status in African American and Nigerian combined samples.**
(DOC)

## References

[1] Siegel R, Naishadham D, Jemal A (2012). Cancer statistics, 2012.. CA Cancer J Clin.

[2] Jemal A, Bray F, Forman D, O’Brien M, Ferlay J (2012). Cancer burden in Africa and opportunities for prevention.. Cancer.

[3] Bernstein L, Ross RK (1993). Endogenous hormones and breast cancer risk.. Epidemiol Rev.

[4] Hankinson SE, Eliassen AH (2010). Circulating sex steroids and breast cancer risk in premenopausal women.. Horm Cancer.

[5] Russo J, Russo IH (2004). Genotoxicity of steroidal estrogens.. Trends Endocrinol Metab.

[6] Maggiolini M, Donze O, Jeannin E, Ando S, Picard D (1999). Adrenal androgens stimulate the proliferation of breast cancer cells as direct activators of estrogen receptor alpha.. Cancer Res.

[7] Gemayel R, Vinces MD, Legendre M, Verstrepen KJ (2010). Variable tandem repeats accelerate evolution of coding and regulatory sequences.. Annu Rev Genet.

[8] Palazzolo I, Gliozzi A, Rusmini P, Sau D, Crippa V (2008). The role of the polyglutamine tract in androgen receptor.. J Steroid Biochem Mol Biol.

[9] Wang W, John EM, Ingles SA (2005). Androgen receptor and prostate-specific antigen gene polymorphisms and breast cancer in African-American women.. Cancer Epidemiol Biomarkers Prev.

[10] Becherini L, Gennari L, Masi L, Mansani R, Massart F (2000). Evidence of a linkage disequilibrium between polymorphisms in the human estrogen receptor alpha gene and their relationship to bone mass variation in postmenopausal Italian women.. Hum Mol Genet.

[11] Fytili P, Giannatou E, Papanikolaou V, Stripeli F, Karachalios T (2005). Association of repeat polymorphisms in the estrogen receptors alpha, beta, and androgen receptor genes with knee osteoarthritis.. Clin Genet.

[12] Lamp M, Peters M, Reinmaa E, Haller-Kikkatalo K, Kaart T (2011). Polymorphisms in ESR1, ESR2 and HSD17B1 genes are associated with fertility status in endometriosis.. Gynecol Endocrinol.

[13] Edwards A, Hammond HA, Jin L, Caskey CT, Chakraborty R (1992). Genetic variation at five trimeric and tetrameric tandem repeat loci in four human population groups.. Genomics.

[14] Wedren S, Lovmar L, Humphreys K, Magnusson C, Melhus H (2004). Oestrogen receptor alpha gene haplotype and postmenopausal breast cancer risk: a case control study.. Breast Cancer Res.

[15] Iobagiu C, Lambert C, Normand M, Genin C (2006). Microsatellite profile in hormonal receptor genes associated with breast cancer.. Breast Cancer Res Treat.

[16] Tsezou A, Tzetis M, Gennatas C, Giannatou E, Pampanos A (2008). Association of repeat polymorphisms in the estrogen receptors alpha, beta (ESR1, ESR2) and androgen receptor (AR) genes with the occurrence of breast cancer.. Breast.

[17] Zheng W, Long J, Gao YT, Li C, Zheng Y (2009). Genome-wide association study identifies a new breast cancer susceptibility locus at 6q25.1.. Nat Genet.

[18] Cai Q, Wen W, Qu S, Li G, Egan KM (2011). Replication and functional genomic analyses of the breast cancer susceptibility locus at 6q25.1 generalize its importance in women of chinese, Japanese, and European ancestry.. Cancer Res.

[19] Chen F, Chen GK, Millikan RC, John EM, Ambrosone CB (2011). Fine-mapping of breast cancer susceptibility loci characterizes genetic risk in African Americans.. Hum Mol Genet.

[20] Hutter CM, Young AM, Ochs-Balcom HM, Carty CL, Wang T (2011). Replication of breast cancer GWAS susceptibility loci in the Women’s Health Initiative African American SHARe Study.. Cancer Epidemiol Biomarkers Prev.

[21] Huo D, Zheng Y, Ogundiran TO, Adebamowo C, Nathanson KL (2012). Evaluation of 19 susceptibility loci of breast cancer in women of African ancestry.. Carcinogenesis.

[22] Stacey SN, Sulem P, Zanon C, Gudjonsson SA, Thorleifsson G (2010). Ancestry-shift refinement mapping of the C6orf97-ESR1 breast cancer susceptibility locus.. PLoS Genet.

[23] Forsti A, Zhao C, Israelsson E, Dahlman-Wright K, Gustafsson JA (2003). Polymorphisms in the estrogen receptor beta gene and risk of breast cancer: no association.. Breast Cancer Res Treat.

[24] Anghel A, Raica M, Marian C, Ursoniu S, Mitrasca O (2006). Combined profile of the tandem repeats CAG, TA and CA of the androgen and estrogen receptor genes in breast cancer.. J Cancer Res Clin Oncol.

[25] Lin PI, Vance JM, Pericak-Vance MA, Martin ER (2007). No gene is an island: the flip-flop phenomenon.. Am J Hum Genet.

[26] Calvo RM, Asuncion M, Sancho J, San Millan JL, Escobar-Morreale HF (2000). The role of the CAG repeat polymorphism in the androgen receptor gene and of skewed X-chromosome inactivation, in the pathogenesis of hirsutism.. J Clin Endocrinol Metab.

[27] Cox DG, Blanche H, Pearce CL, Calle EE, Colditz GA (2006). A comprehensive analysis of the androgen receptor gene and risk of breast cancer: results from the National Cancer Institute Breast and Prostate Cancer Cohort Consortium (BPC3).. Breast Cancer Res.

[28] Rebbeck TR, Kantoff PW, Krithivas K, Neuhausen S, Blackwood MA (1999). Modification of BRCA1-associated breast cancer risk by the polymorphic androgen-receptor CAG repeat.. Am J Hum Genet.

[29] Spurdle AB, Dite GS, Chen X, Mayne CJ, Southey MC (1999). Androgen receptor exon 1 CAG repeat length and breast cancer in women before age forty years.. J Natl Cancer Inst.

[30] Dunning AM, McBride S, Gregory J, Durocher F, Foster NA (1999). No association between androgen or vitamin D receptor gene polymorphisms and risk of breast cancer.. Carcinogenesis.

[31] Young IE, Kurian KM, Mackenzie MA, Kunkler IH, Cohen BB (2000). The CAG repeat within the androgen receptor gene in male breast cancer patients.. J Med Genet.

[32] Given HF, Radbourne R, Oag H, Merritt S, Barclay E (2000). The androgen receptor exon 1 trinucleotide repeat does not act as a modifier of the age of presentation in breast cancer.. Eur J Cancer.

[33] Yu H, Bharaj B, Vassilikos EJ, Giai M, Diamandis EP (2000). Shorter CAG repeat length in the androgen receptor gene is associated with more aggressive forms of breast cancer.. Breast Cancer Res Treat.

[34] Menin C, Banna GL, De Salvo G, Lazzarotto V, De Nicolo A (2001). Lack of association between androgen receptor CAG polymorphism and familial breast/ovarian cancer.. Cancer Lett.

[35] Kadouri L, Easton DF, Edwards S, Hubert A, Kote-Jarai Z (2001). CAG and GGC repeat polymorphisms in the androgen receptor gene and breast cancer susceptibility in BRCA1/2 carriers and non-carriers.. Br J Cancer.

[36] Giguere Y, Dewailly E, Brisson J, Ayotte P, Laflamme N (2001). Short polyglutamine tracts in the androgen receptor are protective against breast cancer in the general population.. Cancer Res.

[37] Elhaji YA, Gottlieb B, Lumbroso R, Beitel LK, Foulkes WD (2001). The polymorphic CAG repeat of the androgen receptor gene: a potential role in breast cancer in women over 40.. Breast Cancer Res Treat.

[38] Kristiansen M, Langerod A, Knudsen GP, Weber BL, Borresen-Dale AL (2002). High frequency of skewed X inactivation in young breast cancer patients.. J Med Genet.

[39] Haiman CA, Brown M, Hankinson SE, Spiegelman D, Colditz GA (2002). The androgen receptor CAG repeat polymorphism and risk of breast cancer in the Nurses’ Health Study.. Cancer Res.

[40] Dagan E, Friedman E, Paperna T, Carmi N, Gershoni-Baruch R (2002). Androgen receptor CAG repeat length in Jewish Israeli women who are BRCA1/2 mutation carriers: association with breast/ovarian cancer phenotype.. Eur J Hum Genet.

[41] Suter NM, Malone KE, Daling JR, Doody DR, Ostrander EA (2003). Androgen receptor (CAG)n and (GGC)n polymorphisms and breast cancer risk in a population-based case-control study of young women.. Cancer Epidemiol Biomarkers Prev.

[42] Syrjakoski K, Hyytinen ER, Kuukasjarvi T, Auvinen A, Kallioniemi OP (2003). Androgen receptor gene alterations in Finnish male breast cancer.. Breast Cancer Res Treat.

[43] Liede A, Zhang W, De Leon Matsuda ML, Tan A, Narod SA (2003). Androgen receptor gene polymorphism and breast cancer susceptibility in The Philippines.. Cancer Epidemiol Biomarkers Prev.

[44] MacLean HE, Brown RW, Beilin J, Warne GL, Zajac JD (2004). Increased frequency of long androgen receptor CAG repeats in male breast cancers.. Breast Cancer Res Treat.

[45] Spurdle AB, Antoniou AC, Duffy DL, Pandeya N, Kelemen L (2005). The androgen receptor CAG repeat polymorphism and modification of breast cancer risk in BRCA1 and BRCA2 mutation carriers.. Breast Cancer Res.

[46] Slattery ML, Sweeney C, Herrick J, Wolff R, Baumgartner K (2007). ESR1, AR, body size, and breast cancer risk in Hispanic and non-Hispanic white women living in the Southwestern United States.. Breast Cancer Res Treat.

[47] Gonzalez A, Javier Dorta F, Rodriguez G, Brito B, Rodriguez MA (2007). Increased risk of breast cancer in women bearing a combination of large CAG and GGN repeats in the exon 1 of the androgen receptor gene.. Eur J Cancer.

[48] Wedren S, Magnusson C, Humphreys K, Melhus H, Kindmark A (2007). Associations between androgen and Vitamin D receptor microsatellites and postmenopausal breast cancer.. Cancer Epidemiol Biomarkers Prev.

[49] Wu MH, Chou YC, Yu CP, Yang T, You SL (2008). Androgen receptor gene CAG repeats, estrogen exposure status, and breast cancer susceptibility.. Eur J Cancer Prev.

[50] Abbas S BL, Chang-Claude J, Hein R, Kropp S, Parthimos M (2010). Polymorphisms in genes of the steroid receptor superfamily modify postmenopausal breast cancer risk associated with menopausal hormone therapy.. Int J Cancer.

[51] Chintamani, Kulshreshtha P, Chakraborty A, Singh L, Mishra AK (2010). Androgen receptor status predicts response to chemotherapy, not risk of breast cancer in Indian women.. World J Surg Oncol.

[52] Sakoda LC, Blackston CR, Doherty JA, Ray RM, Lin MG (2011). Selected estrogen receptor 1 and androgen receptor gene polymorphisms in relation to risk of breast cancer and fibrocystic breast conditions among Chinese women.. Cancer Epidemiol.

[53] Hietala M, Henningson M, Torngren T, Olsson H, Jernstrom H (2011). Androgen receptor htSNPs in relation to androgen levels and OC use in young women from high-risk breast cancer families.. Mol Genet Metab.

[54] Rajender S, Francis A, Pooja S, Krupakar N, Surekha D (2011). CAG repeat length polymorphism in the androgen receptor gene and breast cancer risk: data on Indian women and survey from the world.. Breast Cancer Res Treat.

[55] Mehdipour P, Pirouzpanah S, Kheirollahi M, Atri M (2011). Androgen receptor gene CAG repeat polymorphism and breast cancer risk in Iranian women: a case-control study.. Breast J.

[56] Gilbert SF, Soliman AS, Karkouri M, Quinlan-Davidson M, Strahley A (2011). Clinical profile, BRCA2 expression, and the androgen receptor CAG repeat region in Egyptian and moroccan male breast cancer patients.. Breast Dis.

[57] Gilbert SF, Soliman AS, Iniesta M, Eissa M, Hablas A (2011). Androgen receptor polyglutamine tract length in Egyptian male breast cancer patients.. Breast Cancer Res Treat.

