# Supplementary material for: Microsatellites in the Estrogen Receptor (ESR1, ESR2) and Androgen Receptor (AR) Genes and Breast Cancer Risk in African American and Nigerian Women
Source: PLoS One. 2012 Jul 11;7(7):e40494. doi: 10.1371/journal.pone.0040494 (PMC3394707; doi:10.1371/journal.pone.0040494)
Supplement: Table S1 — Continuous variable analysis of ESR1_TA, ESR2_CA, and AR_CAG alleles and breast cancer risk stratified by ER status in African American and Nigerian women. (DOC) [file pone.0040494.s001.doc]

**Table S1.** Continuous variable analysis of ESR1_TA, ESR2_CA, and AR_CAG alleles and breast cancer risk stratified by ER status in African American and Nigerian women

|  |  | **Repeat length, Mean ± SD** | | | | | | **Multinomial Logistic Regression *P*, RRR (95% CI)** | |
| --- | --- | --- | --- | --- | --- | --- | --- | --- | --- |
|  |  | **AA** | | | **NG** | | | **AA + NG** | |
| **STR** | **Allele^1^** | **ER+ (n=88)** | **ER- (n=86)** | **Control (n=259)** | **ER+ (n=23)** | **ER- (n=48)** | **Control (n=296)** | **ER+ vs. Control** | **ER- vs. Control** |
| ESR1_TA | S | 15.36 ± 2.84 | 15.04 ± 2.83 | 15.06 ± 2.78 | 14.43 ± 2.59 | 15.08 ± 2.98 | 14.78 ± 2.66 | 0.574, 1.022 (0.948-1.100) | 0.712, 1.013 (0.946-1.085) |
|  | L | 19.40 ± 3.60 | 19.14 ± 3.60 | 19.66 ± 3.63 | 18.65 ± 3.76 | 20.38 ± 3.40 | 19.47 ± 3.30 | 0.398, 0.975 (0.920-1.033) | 0.945, 1.002 (0.949-1.058) |
|  | Ave. | 17.38 ± 2.84 | 17.09 ± 2.79 | 17.36 ± 2.78 | 16.54 ± 2.82 | 17.73 ± 2.51 | 17.13 ± 2.53 | 0.791, 0.990 (0.916-1.069) | 0.816, 1.008 (0.940-1.082) |
| ESR2_CA | S | 21.36 ± 2.31 | 21.33 ± 2.45 | 21.27 ± 3.03 | 22.30 ± 3.14 | 21.77 ± 2.69 | 21.02 ± 2.94 | 0.179, 1.057 (0.975-1.145) | 0.224, 1.045 (0.973-1.122) |
|  | L | 23.97 ± 2.03 | 23.74 ± 1.44 | 24.20 ± 2.05 | 24.52 ± 2.47 | 24.17 ± 1.92 | 23.95 ± 2.02 | 0.983, 0.999 (0.899-1.110) | 0.324, 0.951 (0.860-1.051) |
|  | Ave. | 22.66 ± 1.77 | 22.53 ± 1.64 | 22.74 ± 2.13 | 23.41 ± 2.39 | 22.97 ± 1.86 | 22.49 ± 2.05 | 0.339, 1.054 (0.946-1.174) | 0.698, 1.019 (0.926-1.121) |
| AR_CAG | S | 18.33 ± 2.38 | 18.45 ± 2.73 | 17.83 ± 2.77 | 17.78 ± 2.47 | 17.02 ± 2.26 | 17.73 ± 2.30 | 0.244, 1.049 (0.968-1.138) | 0.678, 1.016 (0.943-1.095) |
|  | L | 21.74 ± 2.65 | 22.01 ± 3.11 | 21.78 ± 2.99 | 21.04 ± 2.55 | 20.94 ± 2.56 | 21.38 ± 2.91 | 0.628, 0.982 (0.914-1.056) | 0.897, 0.996 (0.932-1.063) |
|  | Ave. | 20.03 ± 2.21 | 20.23 ± 2.50 | 19.81 ± 2.42 | 19.41 ± 2.24 | 18.98 ± 1.90 | 19.56 ± 2.22 | 0.729, 1.016 (0.929-1.110) | 0.884, 1.006 (0.927-1.092) |

^1^ S: Short allele; L: Long allele; Ave.: average repeat length of short and long alleles
