# Supplementary material for: Microsatellites in the Estrogen Receptor (ESR1, ESR2) and Androgen Receptor (AR) Genes and Breast Cancer Risk in African American and Nigerian Women
Source: PLoS One. 2012 Jul 11;7(7):e40494. doi: 10.1371/journal.pone.0040494 (PMC3394707; doi:10.1371/journal.pone.0040494)
Supplement: Table S2 — Categorical variable analysis of ESR1_TA, ESR2_CA, and AR_CAG alleles and breast cancer risk stratified by ER status in African American and Nigerian combined samples. (DOC) [file pone.0040494.s002.doc]

**Table S2.** Categorical variable analysis of ESR1_TA, ESR2_CA, and AR_CAG alleles and breast cancer risk stratified by ER status in African American and Nigerian combined samples

| **Microsatellite** | **Dichotomous cut-off** | **Genotype^1^** | **Control, n (%)** | **ER+, n (%)** | **ER-, n (%)** | **ER+ vs. Control, RRR (95% CI)** | **Logistic regression *P*** | **ER- vs. Control, RRR (95% CI)** | **Logistic regression *P*** |
| --- | --- | --- | --- | --- | --- | --- | --- | --- | --- |
| ESR1_TA | <18 vs. ≥18 | SS | 172 (31.16) | 38 (34.23) | 43 (32.33) | 1.0 (ref.) |  | 1.0 (ref.) |  |
|  |  | SL | 302 (54.71) | 52 (46.85) | 67 (50.38) | 0.83 (0.52-1.32) |  | 0.92 (0.60-1.41) |  |
|  |  | LL | 78 (14.13) | 21 (18.92) | 23 (17.29) | 1.21 (0.66-2.24) |  | 1.18 (0.66-2.10) |  |
|  |  | Total | 552 | 111 | 133 |  |  |  |  |
|  |  | LL vs. SS + SL |  |  |  | 1.35 (0.78-2.33) | 0.29 | 1.25 (0.75-2.08) | 0.40 |
|  |  | SL + LL vs. SS |  |  |  | 0.91 (0.58-1.42) | 0.68 | 0.97 (0.64-1.46) | 0.89 |
| ESR2_CA | <23 vs. ≥23 | SS | 50 (9.01) | 10 (9.01) | 9 (6.72) | 1.0 (ref.) |  | 1.0 (ref.) |  |
|  |  | SL | 261 (47.03) | 44 (39.64) | 59 (44.03) | 0.87 (0.40-1.87) |  | 1.28 (0.59-2.75) |  |
|  |  | LL | 244 (43.96) | 57 (51.35) | 66 (49.25) | 1.18 (0.56-2.52) |  | 1.51 (0.70-3.25) |  |
|  |  | Total | 555 | 111 | 134 |  |  |  |  |
|  |  | LL vs. SS + SL |  |  |  | 1.33 (0.88-2.02) | 0.18 | 1.23 (0.84-1.80) | 0.29 |
|  |  | SL + LL vs. SS |  |  |  | 1.02 (0.49-2.12) | 0.96 | 1.39 (0.66-2.92) | 0.38 |
| AR_CAG | <20 vs. ≥20 | SS | 148 (26.71) | 31 (27.93) | 33 (24.63) | 1.0 (ref.) |  | 1.0 (ref.) |  |
|  |  | SL | 292 (52.71) | 54 (48.65) | 73 (54.48) | 0.81 (0.50-1.34) |  | 1.08 (0.68-1.70) |  |
|  |  | LL | 114 (20.58) | 26 (23.42) | 28 (20.90) | 0.91 (0.50-1.65) |  | 1.00 (0.57-1.76) |  |
|  |  | Total | 554 | 111 | 134 |  |  |  |  |
|  |  | LL vs. SS + SL |  |  |  | 1.04 (0.63-1.72) | 0.87 | 0.95 (0.60-1.52) | 0.84 |
|  |  | SL + LL vs. SS |  |  |  | 0.84 (0.53-1.35) | 0.48 | 1.05 (0.68-1.64) | 0.82 |
| AR_CAG | <22 vs. ≥22 | SS | 282 (50.90) | 52 (46.85) | 71 (52.99) | 1.0 (ref.) |  | 1.0 (ref.) |  |
|  |  | SL | 232 (41.88) | 44 (39.64) | 51 (38.06) | 0.97 (0.62-1.52) |  | 0.85 (0.57-1.27) |  |
|  |  | LL | 40 (7.22) | 15 (13.51) | 12 (8.96) | 1.74 (0.88-3.45) |  | 1.09 (0.54-2.21) |  |
|  |  | Total | 554 | 111 | 134 |  |  |  |  |
|  |  | LL vs. SS + SL |  |  |  | 1.77 (0.92-3.39) | 0.09 | 1.18 (0.60-2.33) | 0.64 |
|  |  | SL + LL vs. SS |  |  |  | 1.09 (0.72-1.66) | 0.68 | 0.88 (0.60-1.29) | 0.53 |

^1^ S: Short allele; L: Long allele
